# An extracytoplasmic function sigma factor-dependent periplasmic glutathione peroxidase is involved in oxidative stress response of *Shewanella oneidensis*

**DOI:** 10.1186/s12866-015-0357-0

**Published:** 2015-02-18

**Authors:** Jingcheng Dai, Hehong Wei, Chunyuan Tian, Fredrick Heath Damron, Jizhong Zhou, Dongru Qiu

**Affiliations:** Institute of Hydrobiology, Chinese Academy of Sciences, 7 South Donghu Road, Wuchang District, Wuhan, 430072 China; University of Chinese Academy of Sciences, Beijing, 100049 China; School of Life Sciences and Technology, Hubei University of Engineering, 272 Jiaotong Avenue, Xiaogan, 432000 China; Division of Infectious Diseases and International Health, Department of Medicine, University of Virginia, Charlottesville, VA 22908 USA; Institute for Environmental Genomics and Department of Botany and Microbiology, The University of Oklahoma, Stephenson Research and Technology Center, 101 David L. Boren Blvd, Norman, OK 73019 USA

**Keywords:** Periplasmic glutathione peroxidase, *Shewanella*, ECF sigma factor, Oxidative stress response

## Abstract

**Background:**

Bacteria use alternative sigma factors (σs) to regulate condition-specific gene expression for survival and *Shewanella* harbors multiple ECF (*e*xtra*c*ytoplasmic *f*unction) σ genes and cognate anti-sigma factor genes. Here we comparatively analyzed two of the *rpoE*-like operons in the strain MR-1: *rpoE-rseA-rseB-rseC* and *rpoE2-chrR*.

**Results:**

RpoE was important for bacterial growth at low and high temperatures, in the minimal medium, and high salinity. The *degP/htrA* orthologue, required for growth of *Escherichia coli* and *Pseudomonas aeruginosa* at high temperature, is absent in *Shewanella,* while the *degQ* gene is RpoE-regulated and is required for bacterial growth at high temperature. RpoE2 was essential for the optimal growth in oxidative stress conditions because the *rpoE2* mutant was sensitive to hydrogen peroxide and paraquat. The operon encoding a ferrochelatase paralogue (HemH2) and a periplasmic glutathione peroxidase (PgpD) was identified as RpoE2-dependent. PgpD exhibited higher activities and played a more important role in the oxidative stress responses than the cytoplasmic glutathione peroxidase CgpD under tested conditions. The *rpoE2*-*chrR* operon and the identified regulon genes, including *pgpD* and *hemH2*, are coincidently absent in several psychrophilic and/or deep-sea *Shewanella* strains*.*

**Conclusion:**

In *S. oneidensis* MR-1, the RpoE-dependent *degQ* gene is required for optimal growth under high temperature. The *rpoE2* and RpoE2-dependent *pgpD* gene encoding a periplasmic glutathione peroxidase are involved in oxidative stress responses. But *rpoE2* is not required for bacterial growth at low temperature and it even affected bacterial growth under salt stress, indicating that there is a tradeoff between the salt resistance and RpoE2-mediated oxidative stress responses.

**Electronic supplementary material:**

The online version of this article (doi:10.1186/s12866-015-0357-0) contains supplementary material, which is available to authorized users.

## Background

The γ-proteobacteria *Shewanella* species have two hallmark traits, respiratory versatility and psychrophily [1,2]. Respiratory versatility is characterized by their ability to utilize a series of organic and inorganic electron acceptors, particularly metals and metalloids of Fe(III), Mn(IV), Ur(VI) and the direct electron transfer to electrodes [3,4]. *Shewanella* species harbor a variety of outer membrane and periplasmic *c*-type cytochrome genes expressed for respiration under different environmental conditions. Bacterial gene expression is regulated by a series of transcriptional factors including alternative sigma factors (σ^S^). Sigma factors are a component of bacterial RNA polymerase (RNAP) and determine promoter selectivity of the holoenzyme, thus playing a central role in the regulation of gene expression. Bacteria usually have one housekeeping σ factor (RpoD) and a variable number of alternative σ factors that possess different promoter-recognition properties [5]. The number of alternative σ factors highly varies among bacteria and may be related to their specific habitat, metabolisms, and development [5-9]. *E*xtra*c*ytoplasmic *f*unction (ECF) σ factors are highly regulated factors that control expression of genes and constitute the third pillar of bacterial signal transduction after the one-component and two-component systems [9]. Most ECF σs are sequestered by an anti-sigma factor, which can be deactivated by proteolysis, conformational change, partner switching (including mimicry) or other unknown mechanisms to release the ECF sigma factor from being sequestered [9]. Once the ECF sigma factor is released it can then activate of transcription of regulon genes throughout the genome. ECF sigma factor RpoE and its regulators have been extensively studied in *E. coli* [10-17], *Pseudomonas aeruginosa* [18-22] and *Bacillus subtilis* [6,7]. RpoE regulates a series of extracytoplasmic functions, including synthesis of envelope proteins, outer membrane protein (OMP) modification, cell envelope structure and cell division in *E. coli* [23]. The RpoE counterpart AlgU/T controls the production of a series of pathogenic factors, lipoproteins, and the extracellular polysaccharide alginate in *P. aeruginosa* which causes the mortality and morbidity of patients with cystic fibrosis [24-26].

The sigma factors of *Shewanella* have remained relatively uncharacterized. The genome of *Shewanella oneidensis* MR-1 encodes 10 sigma factors (RpoD, RpoH, RpoS, RpoN, FliA, and five ECF sigma factors RpoE, RpoE2, SO_3551 (ECF-like), SO_3096 (ECF-like) and SO_3840 (ECF-like). Sigma32 (RpoH) is the heat shock response sigma factor and it has been shown that heat shock activates expression of 323 genes and represses expression of 286 genes [27,28]. In *S. violacea* strain DSS12, three RpoE-like sigma factors have been identified [29,30]. Numerous transcriptomic studies have shown *Shewanella* can modulate gene expression in response to its environmental signals [29-37]. To shed light on the role of two of the RpoE sigma factors of *S. oneidensis* MR-1, comparative studies were conducted in this study. Deletion mutants were generated and utilized to ascertain the specific functions of each RpoE sigma factor and the two sigma factors dependent genes were identified. RpoE was required for growth at cold and high temperatures, in minimal media, and in high salt environments. Unlike RpoE, RpoE2 is responsible for resistance to oxidative stress. PgpD was identified as the RpoE2 dependent periplasmic glutathione peroxidase that facilitates resistance to oxidative stress. Understanding the regulation of RpoE and RpoE2 and the genes they control can help explain the ability of *S. oneidensis* to survive against environmental stress.

## Results

### RpoE ECF sigma factors and anti-sigma factor genes in *S. oneidensis* MR-1

**Figure 1:**
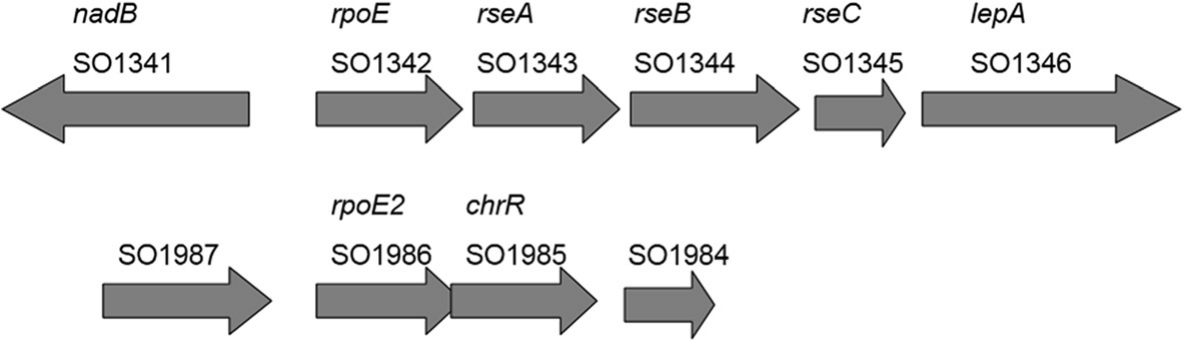
**The gene clusters of** ***rpoE-rseA-rseB-rseC*** **and** ***rpoE2-ChrR*** **and the flanking loci on the chromosome of the** ***S. oneidensis*** **MR-1 strains.** The conserved gene cluster *rpoE-rseA-rseB-rseC* and the flanking genes are also found in the genomes of *Escherichia coli* and *Pseudomonas aeruginosa*.

The homologues for the *E. coli* primary σ factor, RpoD, and five out of six alternative σ factors RpoN, RpoS, RpoH, RpoE, and FliA (RpoF), are present in all the sequenced genomes of *Shewanella* (data not shown)*.* Several *Shewanella* strains such as *S. baltica* OS155 and *S. putrefaciens* W3-18-1 also contain another FliA for lateral flagella [38]. However, the FecR (anti-sigma factor)-FecI (sigma factor)-FecA (ferric citrate receptor) iron-starvation signaling system is absent in most of the sequenced *Shewanella* strains, except for a few *S. baltica* strains. There are five ECF-like σ factors, encoded by SO_1342, SO_1986 SO_3096, SO_3551, and SO_3840, found in *S. oneidensis* MR-1. SO_1342 was identified as the orthologue for *rpoE* (σ^E^) of *E. coli* and *algU/T* of *P. aeruginosa* based on the high sequence similarity and the well-conserved gene cluster of *rpoE-rseA-rseB-rseC* and flanking genes (Figure 1). SO_1986 (designated *rpoE2* hereafter) encodes the orthologue for RpoE of the photosynthetic α-proteobacterium *Rhodobacter sphaeroides*, and the downstream locus SO_1985 encodes the putative cognate anti-σ factor homologous to ChrR [39]. We further characterized the cellular functions of *rpoE* (σ^E^) and *rpoE2* experimentally and computationally.

### RpoE and RpoE2 of *S. oneidensis* are responsible for diverse stress responses

**Figure 2:**
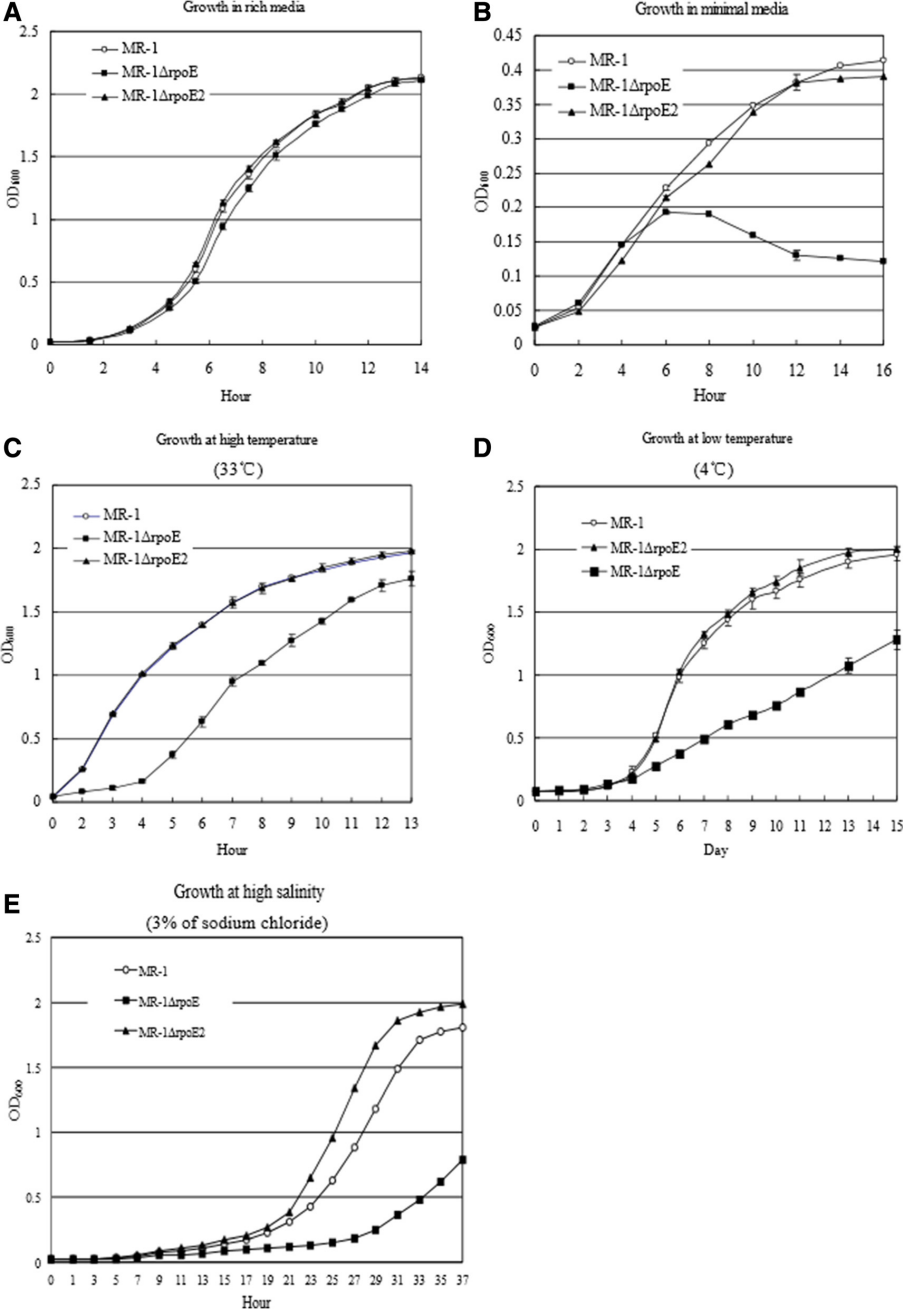
**The** ***rpoE*** **mutant had growth defects when cultured in minimal media, high salinity, and high or low temperature.** Bacterial growth, as measured by OD_600_, are show for the strains growing in various conditions: **A)** Rich medium (LB broth); **B)** Nutrient-poor environment (the modified M1 minimal medium); **C)** Higher temperature (at 33°C and in the LB medium), **D)** Low temperature (at 4°C and in the LB medium); **E)** High salt stress (LB medium supplemented with 3% of sodium chloride, w/v).

In order to characterize the roles of each of the RpoE sigma factors, the *rpoE* and *rpoE2* genes were deleted from strain MR-1. The *rpoE* and *rpoE2* mutant strains had no observable growth defects in rich media (Figure 2A). We examined the role of the *rpoE* sigma factor genes in growth under the stress conditions. The *rpoE* mutant displayed a severe growth defect when cultured in the minimal medium, but no growth defect was observed for the *rpoE2* mutant strain (Figure 2B). As expected the *rpoE* mutant had a growth defect at high temperature (33°C) however no growth defect was observed for the *rpoE2* mutant (Figure 2C). In addition, the *rpoE* mutant also showed a growth defect at low temperatures (4°C, Figure 2D and 10°C, Additional file 1: Figure S1) and high salinity (LB medium supplemented with 3% of sodium chloride, w/v) (Figure 2E). Though the *rpoE* mutant showed an apparent growth defect at high salinity, the growth of the *rpoE2* mutant was even better than that of the wild type strain (Figure 2E and Additional file 1: Figure S1). The *rpoE* mutant was susceptible to ampicillin (data not shown), though the *S. oneidensis* MR-1 wild type strain is resistant to this antibiotic [40].

### RpoE is autoreglated and DegQ is RpoE-dependent

**Figure 3:**
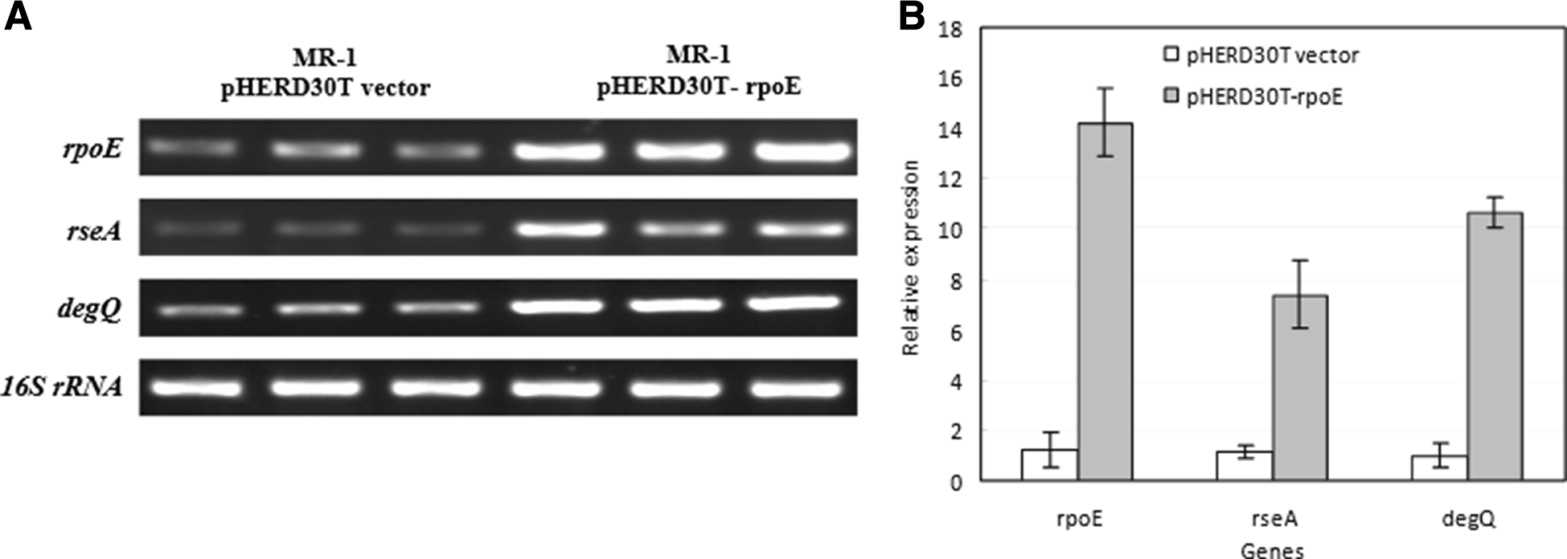
**Expression of the** ***rpoE*** **in the** ***Shewanella oneidensis*** **MR-1 activates expression of** ***rpoE*** **,** ***rseA*** **, and** ***degQ*** **.** Strain MR-1 carrying the pHERD30T-*rpoE* plasmid was grown in the presense of L-arabinose (0.05% w/v). The MR-1 strain carrying only empty pHERD30T (with the pBAD promoter) vector was used as control. Note that the transcripts of *rpoE* in the treatment (the right lanes) include the *in trans* expression of plasmid-borne *rpoE* gene, which further enhances the expression of chromosomal loci of *rpoE* (SO_1342), *rseA* (SO_1343), and *degQ* (SO_3942). The transcription of the chromosomal *rpoE* gene and down-stream cognate anti-sigma factor gene *rseA* is driven by the same promoter, and an RpoE-recognized promoter for autoregulation has been identified upstream of the *rpoE-rseA-rseB-rseC* operon. The cells were collected for RNA extraction after 1 hour of induction. **A)** Transcription of the genes was examined by using semi-quantitative RT-PCR; 16S rRNA gene expression was analyzed and used as the loading control. **B)** Trace quantity plotting of Figure 3A using ‘Quantity One’ software. The assays were performed in triplicates.

The multiple aligment and sequence logos analyses of promoter sequences upstream of *rpoE/algU* in *Shewanella oneidensis*, *Pseudomonas aeruginosa* and *Escherichia coli* were shown the conserved −35 and −10 motifs GAACTT---16/17 bp---TCCAAA upstream of *rpoE/algU* (Additional file 1: Figure S2). By using the Clustal W multiple alignment and Weblogo software[23], we also identified two conserved motifs GAACTT and TCTACA upstream of *rpoE* in 17 *Shewanella* strains, which are similar to the −35 and −10 consensus sequences of the RpoE-dependent promoter (Additional file 1: Table S3)*.* Furthermor, we mapped the transcription start site (TSS) of *rpoE* (SO_1342) by using primer extension (Additional file 1: Figure S3A). In addition, expression of the pHERD30T-*rpoE in trans* did significantly enhance the transcription of chromosomal *rseA* locus in the MR-1 strain, indicating that the expression of the *rpoE-rseABC* gene cluster could be up-regulated by RpoE (i.e., autoregulation, Figure 3).

**Figure 4:**
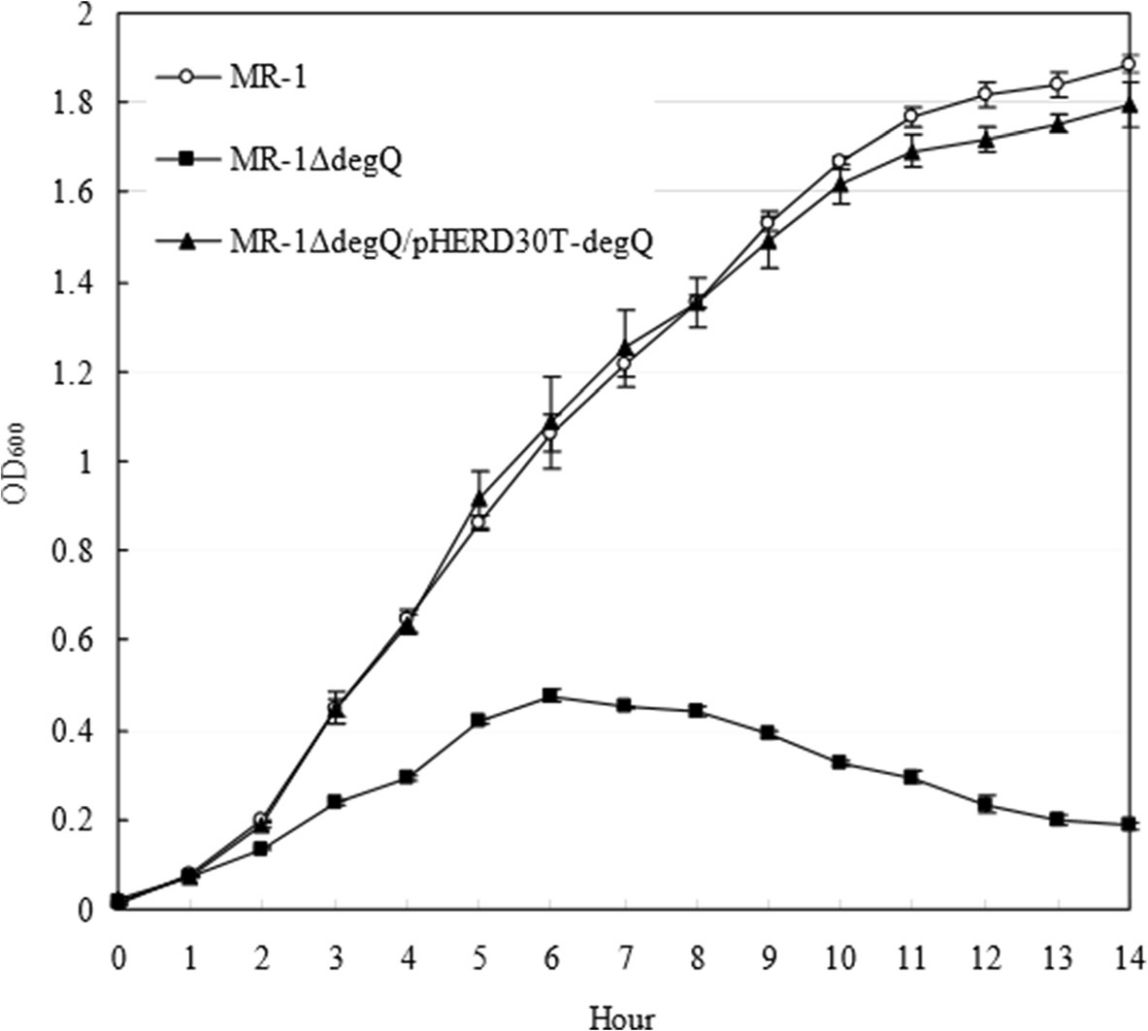
**DegQ is required for optimal growth of strain MR-1 under high temperature.** Genetic complementation by plasmid-borne *degQ* rescued bacterial growth of the *degQ* mutant. The MR-1ΔdegQ strains carrying empty vector (labeled as MR-1ΔdegQ) and pHERD30T-*degQ* plasmid (MR-1ΔdegQ+*degQ*) and the pHERD30T-carrying wild type MR-1 (MR-1) strains were grown in LB broth supplemented with 15 μg/ml of gentamycin. Bacterial strains were grown at 35°C.

Based on the promoter motif recognition and the knowledge on *E. coli* and *P. aeruginosa*, part of the RpoE-dependent regulon was predicted in the genome of MR-1 (Table 1). These genes encode the OMP assembly complex BamABCDE and the lipopolysaccharide (LPS) assembly complex components LptABCD and lipid biosynthesis-related proteins LpxA, B, and D. The *fkpA*, *surA*, *skp* and *ppiA* genes are involved in the proper folding of OMPs [17]. The expression of *degP/mucD* gene, encoding the periplasmic protease Do, is RpoE/AlgU-dependent and is required for survival at high temperatures and envelope integrity in *E. coli* and *Pseudomonas* because DegP/MucD could scavenge abnormal proteins in the periplasm and function as a chaperone for assembly of OMPs [23,24]. However, the only one DegP/HtrA homolog (encoded by SO_3942) was identified as the *E. coli* DegQ orthologue other than DegP because it lacks the characteristic Q-linker (residues 55–79) of the latter (Additional file 1: Figure S4) [41,42]. In addition, this gene (*degQ*) is chromosomally linked with *degS* (SO_3943) as previously found in *E. coli*. The deletion of *degQ* also resulted in the susceptibility of *S. oneidensis* MR-1 to ampicillin (data not shown). The *degQ* gene does not belong to RpoE regulon in *E. coli* [23,43] and is absent in *Pseudomonas*. We found that the *degQ* gene was also RpoE-regulated in MR-1 because the induced expression of *rpoE* enhanced the transcription of *degQ* in turn (Figure 3). There is a TA rich region followed by the GAACTT motif upstream of the open reading frame of *degQ* [27]. The heat shock sigma 32 factor gene (*rpoH*) is also regulated by RpoE in MR-1 (Table 1). DegQ might act as a major protease for protein quality control in the periplasm in the absence of DegP. Deletion of *degQ* resulted in severe growth defectiveness under a higher temperature and the growth of mutant could be rescued by genetic complementation of plasmid borne-*degQ* gene (Figure 4). These results showed that *degQ* played a central role in the high temperature growth of *Shewanella* in the absence of the RpoE-dependent protease Do (DegP).

**Table 1:** **Promoter motifs-based prediction of RpoE and RpoE2 regulon members in the genome of** ***Shewanella oneidensis*** **MR-1**

**Gene identity**	**Putative RpoE-dependent promoter sequence**	**Gene name**	**Functions**	**Other genes in the operon**
SO_0516	**GAACTT**ATGTTTAAAATGACT**GTCAGA**		Hypothetical protein	
SO_1065	**GAACTT**GCTCCTAAAGTTGGT**GTCTCT**	*fkpA*	FKBP-type peptidyl prolyl *cis*-trans isomerase	
SO_1342	**GAACTT**TTTCAAAGTACGCGA**GTCTAC**	*rpoE*	RNA polymerase sigma 24 factor	*rseA*(SO_1343)-*rseB*(SO_1344)-*rseC*(SO_1345)
SO_1476	**GAACTA**AAACCCGCGGCTTAG**GTCGAA**	*bamE*	Outer membrane protein (OMP) assembly complex subunit E	
SO_1492	**GAACTT**CTCTTCACACCTCGC**CACTAT**	*ppiA*	Peptidyl prolyl cis-trans isomerase A	
SO_1636	**GAACCT**TTAGATTTTTTCAAA**GTCGGA**	*rseP*	Membrane associated zinc metalloprotease	*bamA*(SO_1637)-*skp*(SO_1638)- *lpxD*(SO_1639)-*fabZ*(SO_1640)- *lpxA*(SO_1641)-*lpxB*(SO_1642)- *rnhB*(SO_1643)
SO_1880	**GAACTT**TCTGAGCAATGTCATG**GTCTGT**	*bamC*	OMP assembly complex subunit C	
SO_3309	**GAACTC**AAAGGCGACTTCTTT**GTTCGT**	*bamB*	OMP assembly complex subunit B	
SO_3580	**GAACCG**TACCCGCGTTTTGGG**GTCCAA**	*bamD*	OMP assembly complex subunit D	SO_3581
SO_3636	**CAACTT**TCCCCGTCGATACTT**GTCCAG**	*lptD*	Lipopolysaccharide (LPS) transporter subunit D	*surA*(SO_3637)-*pdxA*(SO_3638)-*ksgA*(SO_3639)
SO_3637	**GAACCT**CAACAAGGACTGAGA**GTCCAA**	*surA*	LPS assembly protein	*pdxA*(SO_3638)-*ksgA*(SO_3639)
SO_3942	**GAACTT**TTTCAATGAGGTGCGT**GTCCGA**	*degQ*	Periplasmic serine protease	
SO_3958	**GAACTG**CTATCGATCTACAAT**GTCACC**	*lptC*	LPS transporter (LPT) subunit C	*lptA*(SO_3959)-*lptB*(SO_3960)
SO_3959	**GAACTC**GATCTCAACACTATG**ATAATG**	*lptA*	LPS transporter subunit A	*lptB*(SO_3960)
SO_4562	**GAACTT**TAGCGTGTAAAATCAC**TCTATG**		Conserved hypothetical protein	
SO_4583	**GAACTT**TTGTTCACTTGCAAT**GTCTAT**	*rpoH*	RNA polymerase sigma 32 factor	
	RpoE2-dependent promoter sequence			
SO_1986	**TGATCC**ATTATTCAAAGGGCCA**CGTATT**	*rpoE2*	ECF RNA polymerase	*chrR (SO_1985, anti-sigma factor)*
SO_1987	**TGATCA**AATTCTGATGATGGTA**CGTAAT**	*Lon*	Lon domain protease	
SO_3349	**TGATCC**CTATCGTAGCAAGTTA**CGTAAT**	*pgpD*	Periplasmic glutathione peroxidase	*hemH2* (SO_3348, ferrochelatase)
SO_3386	**TGATCC**TTGTACAAGAATGGTC**CGTAAT**	*ybgA*	Photoreactivation-associated inner membrane protein	*phrB* (SO_3384, deoxyribo-dipyrimidine photolyase) *cfa* (SO_3379, cyclopropane fatty acid synthase)
SO_4169	**TGATCC**TCACAGTGCTGCTATC**CGTAAC**	*phr*	Deoxyribodipyrimidine photolyase-related protein	SO_4170 (CsgA short chain dehydrogenase/reductase)

### RpoE2 mediates resistance to oxidative stress responses

**Figure 5:**
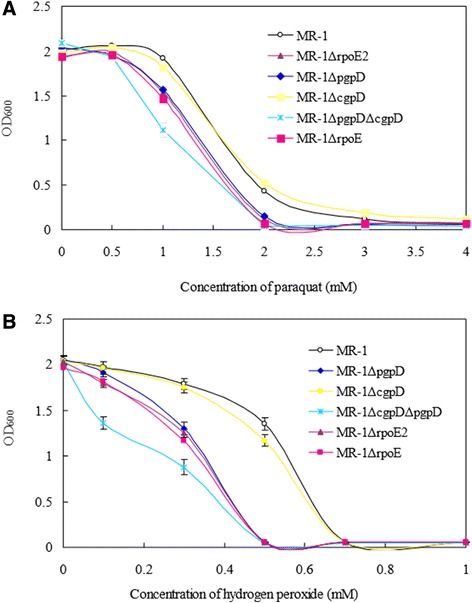
**Effects of paraquat and hydrogen peroxide (H**_**2**_ **O**_**2**_ **) on the bacterial growth of the** ***Shewanella oneidensis*** **strains.** MR-1 wild type strain, the *rpoE2*, *pgpD* (SO_3349) and *cgpD* (SO_1563) in-frame deletion mutants and the *pgpD-cgpD* double mutant strains were grown in the LB broth containing **A)** 0, 0.5, 1, 2, 3, and 4 mM of paraquat or **B)** 0, 0.1, 0.3, 0.5, 0.7 and 1 mM of hydrogen peroxide and incubated at 28°C for 18 hrs.

The *rpoE2-chrR* operon is present in *S. oneidensis* MR-1 (Figure 1 and Additional file 1: Figure S5, S6 and S7) and is absent in *E. coli* and *Pseudomonas*. The open reading frames (ORFs) of *rpoE2* (SO_1986) and *chrR* (SO_1985) are overlapped and the overlapped sequence (*ATG*AT*TAA*) contains the start codon (ATG) of *chrR* and the stop codon (TAA) of *rpoE2*, strongly suggesting that they belong to the same operon and are translationally coupled (Figure 1). The *rpoE2* mutant was more sensitive to hydrogen peroxide and paraquat than the wild type MR-1 strain (Figure 5), indicating that RpoE2 is involved in the oxidative stress responses.

### Identification of the RpoE2 regulon of *S. oneidensis*

**Figure 6:**
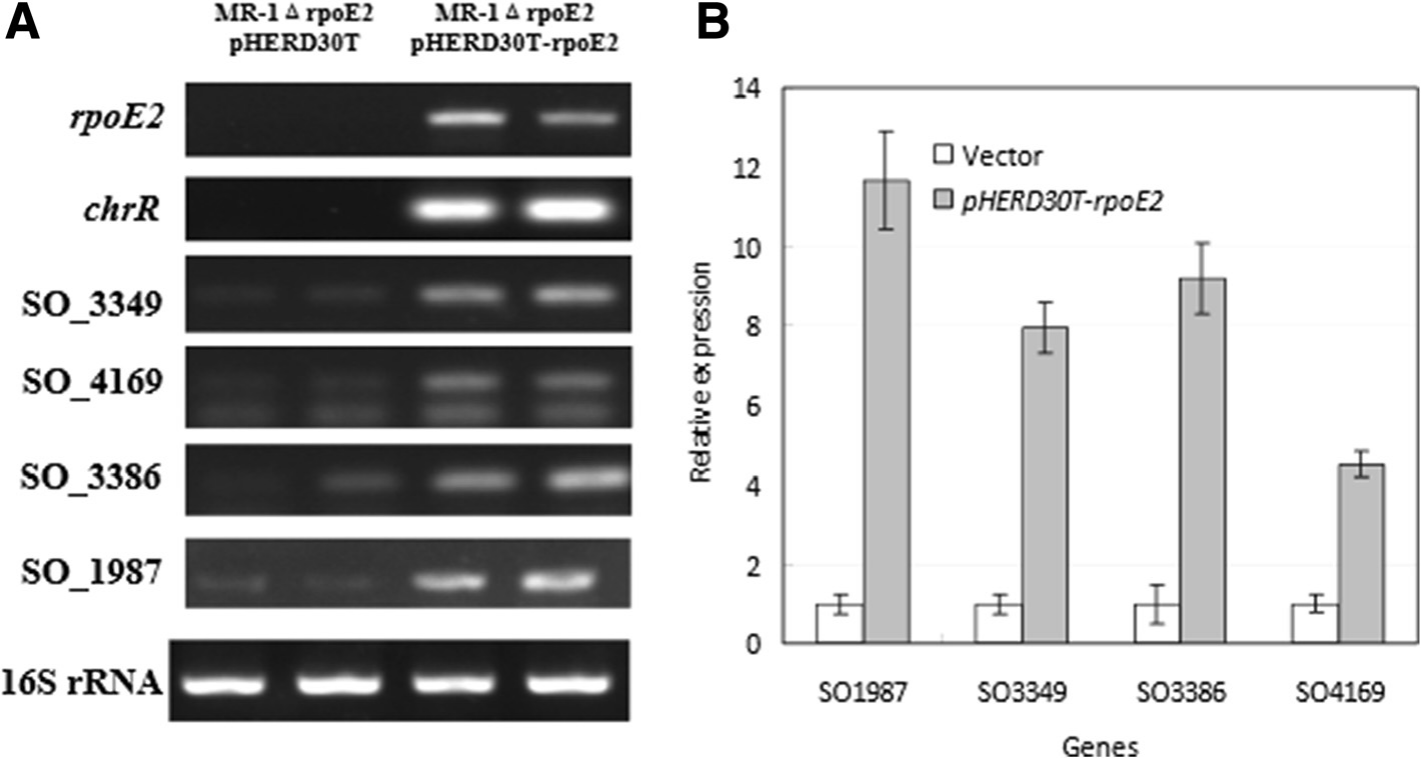
**Induced transcription of the member genes of RpoE2-regulated operons in the** ***rpoE*** **2 null in-frame deletion mutant (MR-1ΔrpoE2) carrying the plasmid-borne** ***rpoE2*** **gene.** The strain carrying pHERD30T empty vector was used as control and 0.01% (w/v) of L-arabinose was added to the bacterial cultures of both control (carrying pHERD30T vector) and treatment (carrying pHERD30T-*rpoE2*) during late exponential phase (OD_600_ > 0.8). The cells were collected for RNA extract after 1 hour of induction. **A)** Transcription of the genes was examined by using semi-quantitative RT-PCR; 16S rRNA gene exp ression was analyzed and used as the loading control. **B)** Trace quantity plotting of figure 6A using ‘Quantity One’ software.The quantitative data represents three times of assays in duplicates.

Multiple alignment analyses on the nucleotide sequences upstream of *rpoE2*-*chrR* revealed two well-conserved motifs, TGATCC and CGTATT, similar to the −35 and −10 elements of RpoE-dependent promoter in *R. sphaeroides* (Additional file 1: Figure S6). Furthermore, we mapped the transcription start site of *rpoE2* (SO_1986) by using primer extension and RT-PCR methods (Additional file 1: Figure S3B and S7). The transcription of *rpoE2* started from A (+1) downstream of the predicted −35 and −10 promoter motifs (Additional file 1: Figure S3B). The core regulon of RpoE2 had been previously predicted based on the promoter consensus sequence in the *Vibrio*-*Shewanella* species [44,45], including *cfa* (SO_3379, encoding cyclopropane fatty acid synthase) and *phrB* (SO_3384, deoxyribodipyrimidine photolyase). The loci SO_3379 (*cfa*) and SO_3384 (*phrB*) obviously belong to the same operon ranging from SO_3386 to SO_3374. By promoter recognition, we were also able to identify other candidates of RpoE2 regulon, including SO_3348 (encoding a ferrochelatase paralogue HemH2 homologous to HemH involved in heme biosynthesis), SO_3349 (a glutathione peroxidase located in the periplasm), SO_4169 (photolyase), SO_4170 (short chain dehydrogenase), and SO_1987 (Lon domain protease) (Additional file 1: Figure S8). These genes probably represent part of the core regulon of RpoE2 coping with photoreactive and oxidative stresses (Additional file 1: Figure S8 and Table 1). Our results also showed that the transcription of *rpoE2* and *chrR* was induced by addition of hydrogen peroxide (3 mM) (Additional file 1: Figure S9). We conducted the semi-quantitative RT-PCR analyses on the RpoE2-induced transcription of several genes of these operons/gene clusters (Figure 6). The L-arabinose induced expression of pHERD30T-borne *rpoE2* remarkably increased the transcription of the chromosomal genes *chrR*, SO_1987, SO_3349, SO_3386, and SO_4169 in the *rpoE2* null mutant. These results indicate that the *rpoE2–chrR* pair is autoregulated and these genes belong to the RpoE2 regulon.

### RpoE2–dependent periplasmic hydrogen peroxidase is involved in oxidative stress response

In light of the fact that RpoE2 plays a role in resistance to oxidative stress, we looked at the RpoE2 regulon for genes that encode proteins that could be responsible. Notably, the RpoE2 regulon member SO_3349 encodes a *p*eriplasmic *g*luthathione *p*eroxidase D (designated *pgpD* hereafter), which may be required for coping with the oxidative stress in the compartment of periplasm. The *pgpD* and the downstream *hemH* paralogue (SO_3348) had not been previously identified as the RpoE-ChrR regulon members in the photosynthetic α-bacterium *Rhodobacter sphaeroides.* The PhoA-fusion assays [46] demonstrated that PgpD is secreted into the periplasm as previously predicted because the signal peptide of PgpD could mediate the secretion of PhoA (Additional file 1: Figure S10). We also mapped the transcription start site of the predicted RpoE2 regulon member SO_3349 and it is shown that the transcription of *pgpD* (SO_3349) does start from the nucleotide A (+1) downstream of the −35 (TGATCC) and −10 (CGTAAT) promoter motifs as it was shown (Additional file 1: Figure S3C and S7). We have generated the in-frame deletion mutants of *pgpD* and *cgpD* and tested the sensitivity of the mutants to hydrogen peroxide and paraquat. Our results showed that the *pgpD* deletion mutant (MR-1ΔpgpD) exhibited a significantly higher sensitivity to oxidative stresses than the MR-1 strain (*p* < 0.01) while no remarkable difference was observed between the *cgpD* mutant (MR-1ΔcgpD) and wild-type strain under the tested concentrations (Figure 5)*.* The growth defectiveness of the *pgpD* deletion mutant in the presence of hydrogen peroxide and paraquat could be rescued by genetic complementation of plasmid borne-*pgpD* gene (Additional file 1: Figure S11). Though PgpD obviously plays a more important role than CgpD under our tested conditions, the double mutant (MR-1ΔcgpDΔpgpD) was more sensitive to hydrogen peroxide stress than the MR-1ΔrpoE2 and MR-1ΔpgpD single mutants (Figure 5), indicating that the *cgpD* gene is also involved in oxidative stress responses.

### Expression and activity assays of cyptoplasmic and periplasmic hydrogen peroxidases

**Figure 7:**
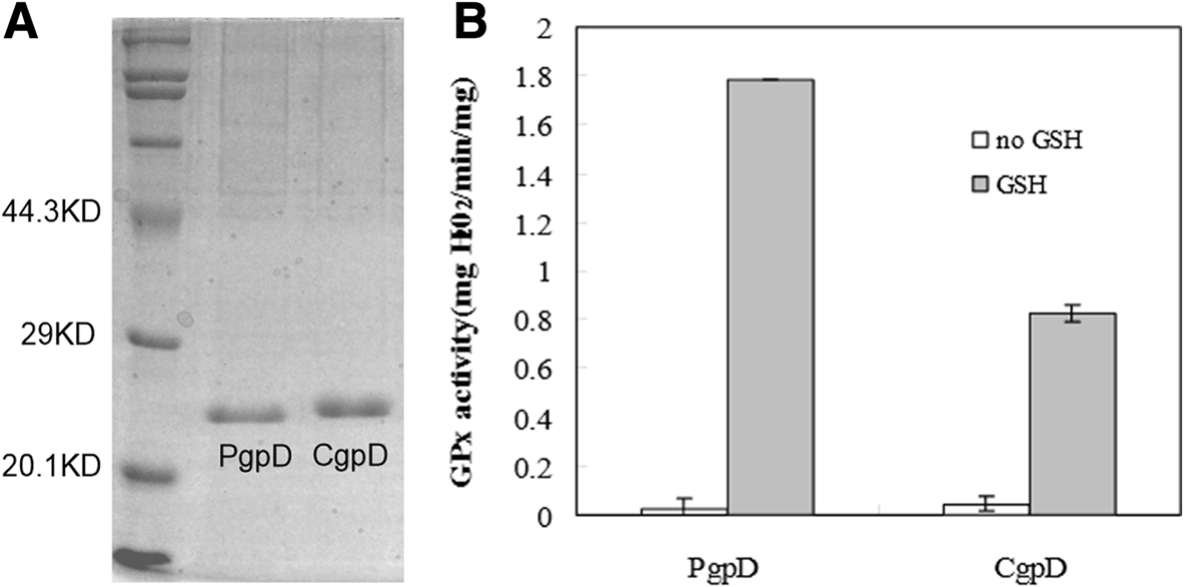
**Glutathione (GSH) peroxidase (GPx) activity assays of PgpD and CgpD. A)** Histidine-tagged CgpD and PgpD proteins were purified. **B)** Glutathione was added and the peroxidase activity is defined as the amount of hydrogen peroxide (mg) broken down in one minute (min) by one mg of purified enzymes (mg H_2_O_2_/min/mg) under the assay conditions described.

The cytoplasmic glutathione peroxidase CgpD and the periplasmic PgpD (residues 20–177) lacking the N-terminal signal peptide (MMKFPLFILTSLMSTSVFA) were successfully overproduced in the *E. coli* BL21/DE3 strain and were purified by Ni-NTP chromatography (Figure 7). Both CgpD and PgpD exhibited the hydrogen peroxide degradation activities in the presence of glutathione (GSH) and the activity of PgpD was higher than that of CgpD under the conditions described (Figure 7). The glutathione export system genes are also present in the genome of MR-1, and are probably involved in the export of GSH from cytoplasm to periplasm. These results, together with the *in vivo* assays (Figure 5), strongly indicated that both PgpD and CgpD were functional in *Shewanella oneidensis* and were probably involved in the degradation of hydrogen peroxide in the periplasm and cytoplasm compartments, respectively.

### Absence of RpoE2-ChrR pair and the regulon members in psychrophilic and/or deep-sea strains

The RpoE2-ChrR system and the regulon members of RpoE2 may play a crucial part in coping with environmental stresses such as UVA radiation and more importantly reactive oxygen species (ROS) in *Shewanella*. The ROS could be sensed by ChrR, which undergoes conformational changes and releases the sequestered RpoE2. The released RpoE2 undergoes auto-upregulation by binding to the promoter of rpoE2-chrR operon and then drives the expression of enzymes involved in modification of cell membrane (Cfa), DNA damage repair (PhrB), degradation of ROS (PgpD) and other stress responses. Our comparative genomic analysis revealed that the *rpoE2-chrR* operon and these identified RpoE2 regulon member genes (SO_1987, SO_3348-SO_3349, SO_3374-SO_3386, and SO_4169-SO_4170) are coincidently absent in several *Shewanella* strains, including *S. pealean* ATCC 700345 [47], *S. sediminis* HAW-EB3 [48], *S. piezotolerans* WP3 [49], *S. halifaxensis* HAW-EB4 [50], *S. violacea* DSS12 [29,51], and *S. benthica* KT99 [52], which are deep-sea and/or psychrophilic strains [53].

## Discussion

In this study the cellular functions of two RpoE-ECF sigma factors of *S. oneidensis* were investigated by comparative genomics, molecular genetics and physiological analyses. We have shown that RpoE is required for bacterial response to a series of stresses, including nutrient depletion (minimal medium), high salinity (3% sodium chloride), high and cold temperatures (33°C and 4°C), and oxidative stresses (hydrogen peroxide and paraquat) in the *S. oneidensis* MR-1 strain. On the other hand, RpoE2 is only involved in oxidative stress responses.

In *E. coli* and *P. aeruginosa*, the *rpoE/algU* gene is autoregulated because an RpoE/AlgU-dependent promoter is located upstream of this gene [23,24]. RpoE regulates a series of extracytoplasmic functions, including synthesis of envelope proteins, outer membrane protein (OMP) modification, cell envelope structure and cell division in *E. coli* [23]. The RpoE counterpart AlgU/T controls the production of a series of pathogenic factors, lipoproteins, and the extracellular polysaccharide alginate in *P. aeruginosa* which causes the mortality and morbidity of patients with cystic fibrosis [24-26]. RpoE is involved in biogenesis of envelope and integrity maintenance as previously demonstrated in mesophilic and psychrophilic bacteria [54]. Our results are consistent with previous microarray analysis data that the *rpoE* exhibited altered transcription under several stress conditions (summarized in the Additional file 1: Table S4). The N-terminal domain of ChrR of *R. sphaeroides* is structurally similar to that of RseA of *E. coli* and defines a common cupin fold among anti-σ factors [44,45].

The *Shewanella* strains harbor a large number of *c*-type cytochrome genes for respiration. A total of 32 and 41 *c*-type cytochrome genes are present in *S. putrefaciens* W3-18-1 and *S. oneidensis* MR-1, respectively [29]. These cytochromes and respiratory chains are a potential source of singlet oxygen [44,45], which may account for the presence of the *rpoE2-chrR* pair and the periplasmic glutathione peroxidase gene *pgpD* in most of the sequenced *Shewanella* strains. On the other hand, the *rpoE2-chrR* pair and the identified regulon members are coincidentally absent in the deep-sea/psychrophilic strains of *Shewanella.* The deep-sea water is characterized by a very low temperature, typically from 0°C to 3°C, a high salinity of about 3.5%, as well as low radiation. As described above, *rpoE2* was not required for bacterial growth under high temperature, nutrient deficiency and particularly cold temperature and high salinity encountered in deep-sea environments. More importantly, the deletion of *rpoE2* even enhanced the bacterial growth under salt stress condition (Figure 2E and Additional file 1: Figure S1). On the other hand, overexpression of *rpoE2* affected bacterial growth under salt stress condition (Additional file 1: Figure S12). These results indicated a tradeoff between oxidative stress response and salt stress tolerance. The loss of these genes may represent a bacterial adaptation to deep-sea and cold environments of high salinity. It remains intriguing why RpoE2–mediated changes affect the bacterial growth under high salinity. The functions and regulation of other ECF σs remain largely unknown in *Shewanella* [30]. The signaling mechanism for the activation and regulon of each σ factor need to be experimentally investigated since their functions could not be completely predicted based on the existing knowledge from the closely related bacteria and comparative genomics analyses as shown by our results.

## Conclusions

Two of the ECF sigma factors, RpoE and RpoE2, regulate a series of extracytoplasmic functions in *S. oneidensis* MR-1. It is revealed that the RpoE-dependent *degQ* gene is required for optimal growth under high temperature. The *rpoE2* and RpoE2-dependent *pgpD* gene are involved in oxidative stress responses. The glutathione peroxidase PgpD is secreted into the periplasm and plays a more important role in oxidative stress responses than the cytoplasmic homlog CgpD. But *rpoE2* is not required for bacterial growth at low temperature and it even affected bacterial growth under salt stress, indicating that there is a tradeoff between the salt resistance and RpoE2-mediated oxidative stress responses.

## Methods

### Bacterial strains, plasmids, culture conditions and genome sequences

The bacterial strains and plasmid used in this study were listed in Additional file 1: Table S1. Bacterial strains were usually cultured in Lysogeny Broth (LB) (containing 10 g tryptone, 5 g yeast extract, and 5 g sodium chloride per litre) media/plates and the modified M1 minimum media (50 mM sodium lactate was used as a carbon source. when necessary, supplemented with 15 and 50 μg/ml of gentamycin and kanamycin, respectively) [32]. *S. oneidensis* MR-1 (ATCC 700550) was isolated from the sediment of Lake Oneida, New York [3] and usually incubated at 28°C in our laboratory. The whole genome sequences for around 30 strains of *Shewanella* are available at the NCBI microbial genome database. The genome of *S. oneidensis* MR-1 was sequenced and annotated by J. Craig Venter Institute [55] and other strains were analyzed by Joint Genome Institute and other institutions*.* The geographical origin of *Shewanella* strains and isolation site characteristics were summarized previously [1,2]. *Pseudomonas aeruginosa* PAO1 strain was obtained from ATCC (Manassas, VA, USA).

### Bioinformatics tools

Polypeptide and nucleotide sequences of genes were retrieved from NCBI database by using BLAST searches. The orthologous relationships among the homologous genes from each bacterial genome are identified by using bidirectional BLASTP searches (best hits) and also based on synteny. The Clustal W package (http://ebi.ac.uk/clustalw) was used for polypeptide and nucleotide sequence alignments and phylogenetic footprinting analyses of promoters and Weblogo (http://weblogo.berkeley.edu) was applied to nucleotide sequence motif identification. The cellular localization of proteins in the cytoplasmic or periplasmic compartment was predicted using Signal P 4.1 server (http://cbs.dtu.dk/services/SignalP).

### Genetic manipulation and genetic complementation

The two-step protocol of selection (gentamycin resistance (Gm^R^) for single cross-over) and counter-selection (sucrose sensitivity for double crossover) was applied for in-frame deletion of specific genes using the suicide vector pDS3.0 (R6K replicon, *sacB*, Gm^R^)-based constructs with a fusion of upstream and downstream sequences as previously described [56]. The genes of *S. oneidensis* MR-1 was PCR amplified and cloned into the pHERD30T shuttle vector, which is suitable for cloning of toxic and tightly regulated genes like ECF sigma factor genes [57]. The resultant constructs and empty vector were transferred into the MR-1 wild type strain and mutants as well as the *P. aeruginosa* PAO1 via conjugation.

### RNA extraction, cDNA synthesis and RT-PCR analysis of gene transcription

Total RNA was extracted by using RNAiso Plus (Takara, Dalian, China) or RNAprep pure Cell/Bacteria Kit (Tiangen Biotech, Beijing, China) and RNA was further purified using DNase I treatment. The integrity of RNA was evaluated by agarose (0.8%) gel electrophoresis. The RNA concentration and purity was measured on a spectrophotometer (Nanodrop Technologies, Wilmington, DE, USA). To prepare cDNA, 2 μg of total RNA was reversely transcribed using PrimeScript® RT reagent Kit with gDNA Eraser (Takara, Dalian, China) and TIANscript RT Kit (TIANGEN BIOTECH (Beijing) CO., LTD.) according to the manufacturer’s protocol. The PCR thermal cycles were: 5 min at 95°C for cDNA denaturation, followed by 27–30 cycles of 30 s at 95°C, 30 s at 51-60°C and 30 s at 72°C. A final elongation step was performed for 10 min at 72°C. RT-PCR products were electrophoresed in a 0.8% agarose gel containing ethidium bromide and visualized by ultraviolet light and Bio-Rad Image software. The data presented are relative mRNA levels normalized against 16S rRNA transcript levels, and the value of the control was set to 1. All the experiments described were performed in triplicates or repeated three times in duplicates to obtain means and standard deviation (SD). The PCR products were also sequenced to confirm amplification of target genes. The primers used were listed in the supplemental materials (Additional file 1: Table S2).

### Determination of transcription start site

Terminal deoxynucleotidyl transferase (TdT, Takara) was used to catalyze the incorporation of single deoxynucleotides (dATPs) into the 3′-OH terminus of cDNA to make the dA-tailed cDNA according to the producer’s protocol. Touchdown and nest PCR was used to amplify the dA-tailed cDNA by using an oligdT (5′-gccagtcTTTTTTTTTTTTTTTTT-3′) primer and a specific primer [58]. The PCR product was cloned into pMD18-T vector (Takara, Dalian, China) for sequencing.

### Hydrogen peroxide and paraquat sensitivity assay

To test the bacterial resistance to hydrogen peroxide (0, 0.1, 0.3, 0.5, 0.7 and 1 mM) and paraquat (0, 0.5, 1, 2, 3, and 4 mM) (1,1-dimethyl-4,4′-bipyridylium dichloride, a powerful propagator of superoxide radicals, Tokyo Chemical Industry Co., Tokyo, Japan) cells were grown overnight in LB broth containing different levels of each chemical and growth was monitored by measure optical density at 600 nm as previously described [59].

### Expression, extraction and activity assays of hydrogen peroxidases

The glutathione hydrogen peroxidase (GPx) genes *pgpD* (lacking the N-terminal sequence encoding the signal peptide) and *cgpD* were cloned into the pET28a vector and the overproducing constructs were transferred into *E. coli* DE3/BL21 cells. The DE3 strains were grown in LB medium (supplemented with 100 μg/L of ampicillin) at 37°C to an OD_600_ of approximately 0.6 and the gene expression was induced by addition of IPTG (0.01%, w/v) at 16°C for 24 hours. The harvested *E. coli* cells were homogenized by applying high pressures (JN-02C low temperature ultra-high pressure continuous flow cell disrupter, Juneng Biol. & Technol. Co., Guangzhou, China) and the His-tagged recombinant proteins were purified by using Ni-NTA Sepharose (GE Healthcare, Waukesha, Wisconsin, USA) affinity chromatography according to the supplier’s protocol. The activity of hydrogen peroxidases is assayed by a widely used protocol with some modifications [60]. 3 ml of the enzyme elute from Ni-NTA Sepharose was mixed with 3 ml of phosphate buffer containing 0.1 M hydrogen peroxide and 0.1 M glutathione (GSH). The reaction was stopped by adding 3 ml of 10% (v/v) sulfuric acid and the residual hydrogen peroxide was titrated against 0.1 M permanganate (KMnO_4_) solution until a faint purple color persisted for at least 30 seconds. The enzyme concentrations were measured by using a total protein assay kit (Jiancheng Biotech., Nanjing, China). The same amounts of boiling-denatured enzyme solutions were used as control.

### Alkaline phosphatase A-fusion assay

To determine the protein cellular location, the 5′-nucleotide sequence, encoding the amino-terminal signal peptide (SP), of the *pgpD* gene was translationally fused with *E. coli phoA* gene with deletion of the sequence encoding the N-terminal signal sequence. This *pgpD-phoA* fusion and *phoA* were cloned into pUCP20T vector for alkaline phosphatase A-fusion assay [46], and the transformants of DH5α were plated on the LB plate containing 40 μg/ml of BCIP (5-Bromo-4-chloro-3-indolyl phosphate p-toluidine, Amresco, Solon, OH, USA) and 100 μg/ml of ampicillin. The construct pUCP20-*phoA*(wt) expressing full-length PhoA was used as positive control and the pUCP20-*phoA*(NSP) expressing the truncated PhoA without N-terminal signal leader sequence as negative control.

## Additional file

Additional file 1:
**Supplemental Tables S1-S5 and Figures S1-S12 associated with this manuscript.**
