# Supplementary material for: An extracytoplasmic function sigma factor-dependent periplasmic glutathione peroxidase is involved in oxidative stress response of Shewanella oneidensis
Source: BMC Microbiol. 2015 Feb 18;15:34. doi: 10.1186/s12866-015-0357-0 (PMC4336711; doi:10.1186/s12866-015-0357-0)
Supplement: Additional file 1: — Supplemental Tables S1-S5 and Figures S1-S12 associated with this manuscript. [file 12866_2015_357_MOESM1_ESM.doc]

Dai et al., 2014. Supplemental Figures and Tables

**Supplemental Table S1** Bacterial strains and plasmids

| Strain | Description | Source or reference |
| --- | --- | --- |
| *Escherichia coli* WM3064 | *thrB1004 pro thi rpsL hsdS lacZDM15 RP4-1360 (araBAD)567* *dapA1341::[erm pir(wt)]* | W. Metcalf |
| *E. coli* TOP10 | *F^-^* *mcrA* Δ(*mrr-hsdRMS-mcrBC*) Φ80*lacZ*DM15 Δ *lacX74 deoR recA1 araD139* Δ(*ara-leu*)*7697 galU galK rpsL* (Sm^r^) *endA1 nupG* | Invitrogen |
| *E. coli* EC100D+ | *F^-^ mcrA Δ(mrr-hsdRMS-mcrBC) φ80dlacZΔM15 ΔlacX74 recA1 endA1 araD139 Δ(ara, leu)7697 galU galK λ- rpsL (Str^R^) nupG pir^+^(DHFR)* | Epicentre Technologies |
| *E. coli* DH5α | *F^-^ endA1 glnV44 thi-1 recA1 relA1 gyrA96 deoR nupG Φ80dlacZΔM15 Δ(lacZYA-argF)U169, hsdR17(r_K_^-^ m_K_^+^) phoA λ*– | Takara |
| *Shewanella oneidensis* MR-1 | Dissimilatory metal-reducing strain isolated from Lake Oneida, New York | Myers and Nealson, 1988 |
| MR-1ΔrpoE | In-frame deletion mutant of *rpoE* gene (SO_1342) | This study |
| MR-1ΔrpoE2 | In-frame deletion mutant of *rpoE_2_* gene (SO_1986) | This study |
| MR-1ΔdegQ | In-frame deletion mutant of *degQ* gene (SO_3942) | This study |
| MR-1ΔpgpD | In-frame deletion mutant of *pgpD* gene (SO_3349) | This study |
| MR-1ΔcgpD | In-frame deletion mutant of *cgpD* gene (SO_1563) | This study |
| MR-1ΔcgpDΔpgpD | In-frame deletion double mutant of *cgpD* and *pgpD* | This study |
| *Pseudomonas aeruginosa* PAO1 | Non-mucoid prototroph | ATCC |
| Plasmid | | |
| pDS3.0 | Suicide vector derived from pCDV224; *Amp^r^, Gm^r^, sacB* | Wan *et al*., 2004 |
| pDS3.0-rpoEko | Suicide plasmid for deletion of *rpoE* | This study |
| pDS3.0-rpoE2ko | Suicide plasmid for deletion of *rpoE_2_* | This study |
| pDS3.0-cgpDko | Suicide plasmid for deletion of *cgpD* | This study |
| pDS3.0-pgpDko | Suicide plasmid for deletion of *pgpD* | This study |
| pET28a | Expression vector with T7lac promoter | Novogene |
| pET28a-pgpD | Overexpression construct of *pgpD* lacking N-terminal sequence encoding signal peptide | This study |
| pET28a-cgpD | Overexpression construct of *cgpD* | This study |
| pHERD30T | Shuttle vector with pBAD promoter, *Gm^r^* | Qiu *et al*., 2008 |
| pHERD30T-rpoE | The *rpoE* gene cloned in pHERD30T, Gm^r^ | This study |
| pHERD30T-rpoE2 | The *rpoE_2_* cloned in pHERD30T, *Gm^r^* | This study |
| pHERD30T-degQ | The *degQ* gene cloned in pHERD30T, Gm^r^ | This study |
| pHERD30T-ompW | The *ompW* gene cloned in pHERD30T, Gm^r^ | This study |
| pHERD30T-ompN | The *ompN* gene cloned in pHERD30T, Gm^r^ | This study |
| pUCP20T | Shuttle vector Amp^r^ | H.P. Schweizer |
| pUCP20-phoA(wt) | The *E. coli phoA* gene cloned in pUCP20T | This study |
| pUCP20-phoA(NSP) | The modified *phoA* gene (phoA(NSP)), without the 5’-sequence encoding the N-terminal signal peptide, cloned in pUCP20T | This study |
| pUCP20-pgpD-phoA fusion | The pgpD-*phoA* fusion gene, with the 5’-sequence encoding the N-terminal signal peptide fused with *phoA(NSP)*, cloned in pUCP20T | This study |

**Supplemental Table S2** Primers used in this study

| Primer | Oligonucleotide sequence (5’-3’) |
| --- | --- |
| rpoE_F | 5'-AGAATTCGGAGAAGTCGGCTCGGATGA-3' |
| rpoE_R | 5'-GGTCGACTTACTCTTCCAGCAAAGGCTG-3' |
| rpoE2_F | 5’-AGAATTCCGAACGGGGTTGAACCTTA-3’ |
| rpoE2_R | 5’-AGTCGACGGGGGTGATGTTTAATCATGT-3’ |
| pgpD_F | 5’- GGAATTCGCAACATGCCCAAGCTATCTTG-3’ |
| pgpD_R | 5’- GCAAGCTTTTACAGCACGGATTCTATTGCCTG-3’ |
| cgpD_F | 5’- GGAATTCATGACATCCACTATCTATCG -3’ |
| cgpD_R | 5’- CGCTCGAGTTACTTATTTAAAAGTGACTCTAT -3’ |
| rpoEko_5O | 5’-CAGAGCTCTACGACGTTGCTCATTCATTTGATC-3’ |
| rpoEko_5I | 5’-GACTGGCTTAGGCTTAGGTCGTCTCTGAAGAGTAAATTCTTATTTTAAG-3’ |
| rpoEko_3I | 5’-AGAGACGACCTAAGCCAGTCATCAAGCTAATCACTTTACTCTG-3’ |
| rpoEko_3O | 5’-GTGAGCTCCCAAGCCATCGCAATACCATC-3’ |
| rpoE2ko_5O | 5'-AGTCGATGCCGGAAATCACTA-3' |
| rpoE2ko_5I | 5'-TGCATCGAGTTGATTGTCGCGCTTTTTGGAGACACAACA-3' |
| rpoE2ko_3I | 5'-GCGACAATCAACTCGATGCACCGTTCGAACATGGCTTCA-3' |
| rpoE2ko_3O | 5'-AGAGCTCTGAAGTGCGCCAATAAGCCT-3' |
| degQko_5O | 5'-CAGAGCTCGCTAATACCACATCGCCAACCT-3' |
| degQko_5I | 5'- GACTGGCTTAGGTCGTCTCTGTGAAGCAAACCAAACGACAGA-3' |
| degQko_3I | 5'- AGAGACGACCTAAGCCAGTCCCAAAGAAATAACGGAACACAT-3' |
| degQko_3O | 5'- GTGAGCTCGTACTGCAACACCACTAAAACC-3' |
| pgpDko_5O | 5'-CAGAGCTCTAGAGCGGTAAAACCACCAG -3' |
| pgpDko_5I | 5'-GACTGGCTTAGGTCGTCTCTTAAGCAGGCAATAGAATCCG-3' |
| pgpDko_3I | 5'-AGAGACGACCTAAGCCAGTCCTGAGTGCAATTTGCGCACT -3' |
| pgpDko_3O | 5'-GTGAGCTCTCTCCTTCGCTGAAACAGGC-3' |
| cgpDko_5O | 5'-CAGAGCTCTATCAGAAGGCCAAAAACACGA-3' |
| cgpDko_5I | 5'-GACTGGCTTAGGTCGTCTCTCGCCGACAACGAAACCAGAAGA-3' |
| cgpDko_3I | 5'-AGAGACGACCTAAGCCAGTCGGGGTTGGATTACCCTGAATGT-3' |
| cgpDko_3O | 5'-GTGAGCTCCGCTACCGTTGCTTTATCGCTG -3' |
| pgpD-F(SP) | 5’-CAGAGCTCGTTCGCCTATCTTGGATC-3’ |
| pgpD-R(SP) | 5’-CAGGCATTTCTGGTGTCCGTGCCGCAAAAACACTGGTG-3’ |
| phoA-F(NSP) | 5’-CACCAGTGTTTTTGCGGCACGGACACCAGAAATGCCTG-3’ |
| phoA-R(NSP) | 5’-GCTCTAGAGTTTTATTTCAGCCCCAGAG-3’ |
| phoA-F(wt) | 5’-GTGAGCTCGTCACGGCCGAGACTTATAG-3’ |
| phoA-F(NSP1) | 5’-CAGAGCTCCCGGACACCAGAAATGCCTG-3’ |
| RT-PCR primers |  |
| SO_1342-F | 5’-AGCATTTTAGATTTGGGAGAAGTC -3' |
| SO_1342-R | 5’-TGTTCACCTGTCTAAATTTAAGGG -3' |
| SO_1343-F | 5’-ATAAGTGGCGTAACTATCATTTG -3' |
| SO_1343-R | 5’-GCTAACTCCTTACTACTGATTGACAGG -3' |
| SO_3942-F | 5’-TTGAAAACTTTATCCAAACCGACG -3' |
| SO_3942-R | 5’-AGCGATTTAAGATCTTTAACCGCA -3' |
| SO_1985-F | 5’-AGAATTCCGAACGGGGTTGAACCTTA -3' |
| SO_1985-R | 5’-AGTCGACGGGGGTGATGTTTAATCATGT -3' |
| SO_1986-F | 5’-CGGGTACAGCATAACCGAGAAG -3' |
| SO_1986-R | 5’-AATCATGTTGTGTCTCCAAAAAGC -3' |
| SO_1987-F | 5’-TCCGATAGGAACTCTGGTGC -3' |
| SO_1987-F | 5’-CGGTTCTTAATAAATCCAGTGTG |
| SO_3386-F | 5’-TTTGATTATGTTCCTATTTGTCCAG -3' |
| SO_3386-R | 5’-TGGTGCCAATCTTGTACTCTGTC -3' |
| SO_3349-F | 5’-GGTGGTGATTGGTTTTCCTTCG -3' |
| SO_3349-R | 5’-TAGGCATTACAGCACGGATTC -3' |
| SO_4169-F | 5’-AGATTTGCGGTGATTTCGGC -3' |
| SO_4169-R | 5’-TCCTGTTTGAGTTGGGGTGGC -3' |
| 16S rRNA-RT-F | 5’-GTTGGAAACGACTGCTAATACC -3' |
| 16S rRNA-RT-R | 5’-GGTCCTTCTTCTGTAGGTAACG -3' |

**Supplemental Table S3** Sequence logos of homologous sequences upstream of *rpoE* in *Shewanella* showing the conserved motifs GAACTT and TCTACA similar to those of the *rpoE* orthologs in *E. coli* and *Pseudomonas aeruginosa*.

| Strains | Homologous sequences upstream of *rpoE-rseA-rseB-rseC* |
| --- | --- |
| *Shewanella* sp. ANA-3 | TTA**GAACTTT**TTCAAAGCACGCTA**GTCTAC**ATAAGTGAATATGATTCGAGCGACTGAGAAATCAGCATTTTAGATTTGGGAGAAGTCGGCTCG |
| *Shewanella* sp. MR-4 | TTA**GAACTTT**TTCAAAGCACGCTA**GTCTAC**ATAAGTGAATGTGATTCGAGCGACTGAGAAATCAGCATTTTAGATTTGGGAGAAGTCGGCTCG |
| *S. putreficiens* CN32/W3-18-1 | TAA**GAACTTT**TTCAAAGCCCGCGA**GTCTAC**ATAAGTGAATATGATTCGAGCGACTGAGAAATCAGCATTTTAGATTTGGGAGAAGTCGGCTCG |
| *Shewanella* sp. MR-7 | TTA**GAACTTT**TTCAAAGCACGCTA**GTCTAC**ATAAGTGAATATGATTCGAGCGACTGAGAAATCAGCATTTTAGATTTGGGAGAAGTCGGCTCG |
| *S. oneidensis* MR-1 | TTA**GAACTTT**TTCAAAGTACGCGA**GTCTAC**ATAAGTGAATATGATTCGAGCGACTGAGAAATCAGCATTTTAGATTTGGGAGAAGTCGGCTCG |
| *S. baltica* strains | TTA**GAACTTT**TTCAAAGTACGCGA**GTCTAC**ATAAGTGAATATGATTCGAGCGACTGAGAAATCAGCATTTTAGATTTAGGAGAAGTCGGCTCG |
| *S. loihica* PV-4 | TTA**GAACTTT**TTCGATGTGGGCTA**GTCTAC**ATATGTGAATATGATTCGAGCGACTGAGAAATCAGCATTTTAGATTTAGGAGTAGTCGGCTCG |
| *S. sediminis* HAW-EB3 | TTA**GAACTTT**TCGGAAGTAAGCTA**GTCTAC**ATATGTGAATATGATTCGAGCGACTGAGAAATCAGCATTTTAGATTTAGGAGTAGTCGGCTCG |
| *S. woodyi* ATCC 51908 | TTA**GAACTTT**TTAGAAGTAAGCTA**GTCTAC**ATATGTGAATATGATTCGAGCGACTGAGAAATCAGCATTTTAGATTTAGGAGTAGTCGGCTCG |
| *S. violacea* DSS12 | TTA**GAACTTT**TTCGAAATTCGTTA**GTCTAC**ATATGTGCATATGATTCGAGCGACTGAGAAATCAGCATTTTAGATTTAGGAGTAGTCGGCTCG |
| *S. benthica* KT99 | TTA**GAACTTT**TTCGAAGTTCGTTA**GTCTAC**ATATGTGCATATGATTCGAGCGACTGAGAAATCAGCATTTTAGATTTAGGAGTAGTCGGCTCG |
| *S. frigidimarina* NCIMB 400 | ATC**GAACTTT**CTTCATATCAGTAG**GTCTAC**TTATATGAACAGGATTCGAGCGACTGAGAAATCAGCATTACAGATTTAGGAGTAGTCGGCTCG |
| *S. denitrificans* OS217 | GCC**GAACTTT**TCCAATGAGCGCCA**GTCTAC**ATAATGTGAATAATGTTCGAGCGACTGAGAAATCAGCATTAGATTTAGGAGTAGTCGGCTCGA |
| *S. halifaxensis* HAW-EB4 | TTA**GAACTTT**TTTGAAACGCGCTA**GTCTAC**ATAAGTGAATATGATTCGAGCGGCTGAGAAATCAGTATTAGATTTAGGAGTAGTCGGCTCGG |
| *S. piezotolerans* WP3 | TTA**GAACTTT**TTTGAAACTCGCTA**GTCTAC**ATAAGTGAATATGATTCGAGCGGCTGAGAAATCAGTATTAGATTTAGGAGTAGTCGGCTCGG |
| *S. amazonensis* SB2B | GCA**GAACTTT**TTCAATATACGCTG**GTCTAC**ATAGGTAGTTATGATTCGAGCGACTGAAAAATCAGTGTGTTAGATTTGGGAGAAGTCGGCTCG |
| *S. pealeana* ATCC 700345 | TTA**GAACTTT**TTTGAAACTCGCTA**GTCTAC**ATAAGTGAATATGATTCGAGCGGCTGAGAAATCAGTATTAGATTTAGGAGTAGTCGGCTCGG |
| Logos of sequences | 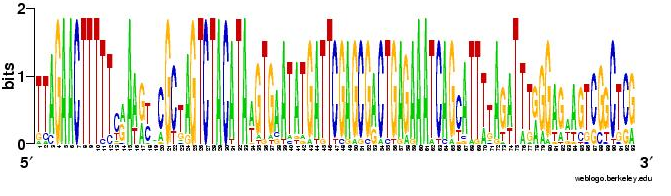 |

**Supplemental Table S4** Transcriptional upregulation (+) and down-regulation (-) of sigma factor (σ) and anti-σ factor genes in *Shewanella oneidensis* MR-1 grown under various stress and inducing conditions

| Sigma factor & anti-σ gene | Stress conditions | | | | | | |
| --- | --- | --- | --- | --- | --- | --- | --- |
|  | UVA | Heat shock | SrCl_2_ shock | Acidic | Alkaline pH | Cr(VI) | K_2_CrO_7_ (1mM) |
|  |  |  |  | pH |  |  |  |
| *rpoE* |  | **+** | **+** |  | **+** | **-** |  |
| *rseA* |  | **+** | **+** |  | **+** | **-** |  |
| *rseB* |  | **+** | **+** |  | **+** | **-** |  |
| *rseC* |  | **+** | **+** |  | **+** | **-** |  |
| *rpoE_2_* | **+** | **+** |  | **-** |  | **-** | **+** |
| *chrR* | **+** | **+** |  | **-** | **-** | **-** | **+** |

**Supplemental Table S5** Orthologs for ECF σ, anti-σ, and the relevant regulatory genes involved in envelope stress responses in *S. oneidensis* MR-1, *G. sulfurreducens* PCA, *E. coli* K12, and *P. aeruginosa* PAO1

| *S. oneidensis* locus | *E. coli* K12 | *P. aeruginosa PAO1* | Gene functions |
| --- | --- | --- | --- |
| SO_1342*, rpoE* | ECK2571*, rpoE* | PA0762*, algT/algU* | ECF σ factor for envelope stress responses |
| SO_1343*, rseA* | ECK2570*, rseA* | PA0763*, mucA* | Cognate anti-σ factor for RpoE |
| SO_1344*, rseB* | ECK2569*, rseB* | PA0764*, mucB* | Negative regulator of RpoE |
| SO_1345*, rseC* | ECK2568*, rseC* | PA0765*, mucC* | Unknown |
| SO_1986*, rpoE_2_* | *-* | *-* | ECF σ factor for reactive oxygen stress |
| SO_1985*, chrR* | *-* | *-* | Cognate anti-σ factor for RpoE_2_ |
| SO_3943*, degS* | ECK3224*, degS* | PA4446*, algW* | Envelope stress sensor serine protease sensing accumulation of abnormal proteins and initiating cleavage of RseA |
| SO_1636*, rseP* | ECK0175*, rseP/yaeL* | PA3649*, mucP* | Inner membrane bound serine protease participating in cleavage of RseA |
| SO_1794*, clpP* | ECK0431*, clpP* | PA1801*, clpP* | Cytoplasmic protease complex ClpXP degrading N-terminus of RseA to release RpoE and other abnormal proteins |
| SO_1795*, clpX* | ECK0432*, clpX* | PA1802*, clpX* | Cytoplasmic ATPase for ClpP |
| *-* | ECK0160*, degP/htrA* | PA0766*, mucD* | Periplasmic serine protease/ chaperone degrading of abnormal proteins for envelope integrity |
| SO_3942*, degQ* | ECK3223*, degQ* | *-* | Periplasmic serine endoprotease degrading transiently denatured proteins |
| SO_2601 & SO_0047 | ECK1829*, prc /tsp* | PA3257 *(prc)* & PA5134 *(tsp)* | Carboxyl-terminal protease (PRC) or tail specific protease (TSP) degrading truncated proteins in the periplasm |

A

B

**Supplemental Figure S1** The bacterial growth of MR-1ΔrpoE2 was enhanced under a high salt and low temperature condition while the population growth of MR-1ΔrpoE was totally inhibited. The bacterial strains were cultured in the LB broth supplemented with sodium chloride (3%, w/v) and incubated in a shaker (200 rpm) set at 10°C (A). No bacterial growth was observed at 4°C (B).

*Pseudomonas*

*Escherichia*

**TTAGAACTTTTTCAAAGTACGCGAGTCTACATAAGTGAATATGATTCGAGCGACTGAG**

**GGAGAACTTTCTTAGACGCATCGGTTCCAAAGCAGGATGCCTGAAGACCTCGTCCGG**

**ACGGAACTTTACAAAAACGAGACACTCTAACCCTTTGCTTGCTCAAATTGCAGCTAAT**

*Shewanella*

**TCTAAAA**

**GAACTTT**

**-35**

**-10**

**
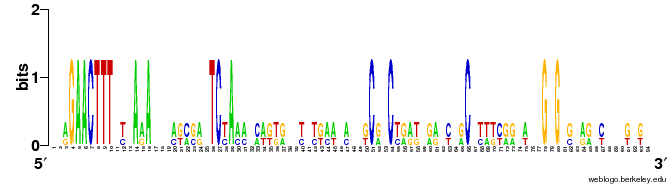
**

**Supplemental Figure S2** The multiple aligment and sequence logos analyses of promoter sequences upstream of *rpoE/algU* in *Shewanella oneidensis*, *Pseudomonas aeruginosa* and *Escherichia coli* were shown in lower panels and the -35 and -10 motifs were highlighted. The consensus sequence is GAACTT---16/17bp---TCCAAA. These results are consistent with the finding that the *rpoE* transcription was auto-regulated in the *S. oneidensis* MR-1 strain (Figure 3 in the main text).

A


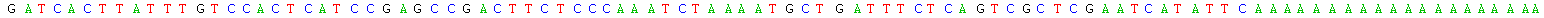


**dATP tailing**

***rpoE* TSS**


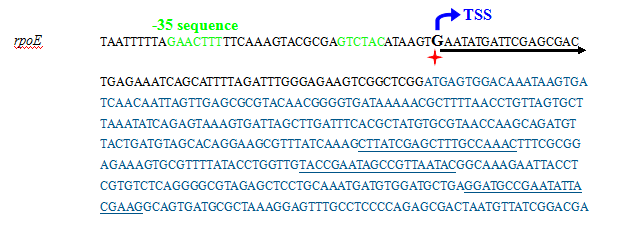


B

**dATP tailing**


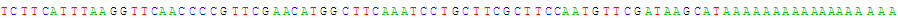


**SO_1986 TSS**


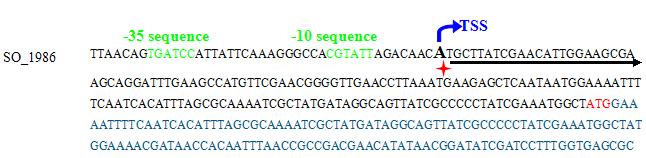


C


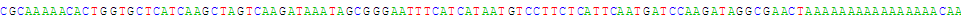


**SO_3349 TSS**

**dATP tailing**


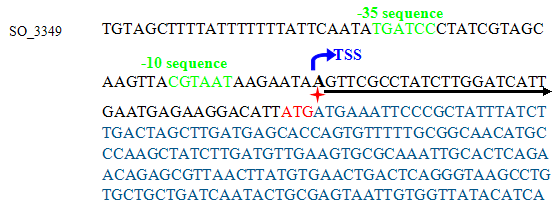


**Supplemental Figure S3 Primer extension analysis of transcriptional start site (TSS) of the *rpoE* (SO_1342, panel A), *rpoE2* (SO_1986, panel B) and *pgpD* (SO_3349, panel C)**. The arrow-pointed nucleotide is the start site of transcription and the predicted -35 and -10 motifs are indicated in green color.


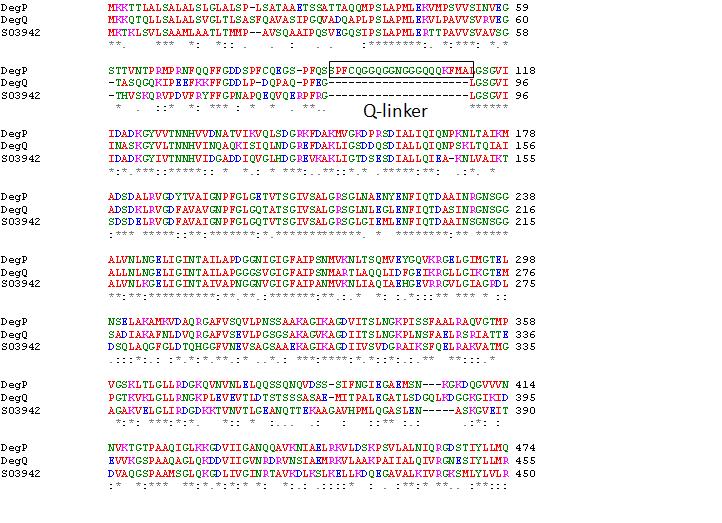


**Supplemental Figure S4** Multiple alignment of the DegP and DegQ of *Escherichia coli* K-12 and the homolog encoded by SO_3942 of *Shewanella oneidensis* MR-1. SO_3942 is considered to be the ortholog of *degQ* of *E. coli* because its gene product lacks the Q-linker and it is chromosomally linked with *degS* as found in *E. coli*.

**SO1985 -MIKHHPHAELLQAHAQGELPLSMSMAVAAHCALCQECQNQIAKLTEQAAKLAFVSSSDD 59**

**RSP1093 MTIRHHVSDALLTAYAAGTLSEAFSLVVATHLSLCDECRARAGALDAVGGSLMEET---A 57**

***:** ** *:* * *. ::*:.**:* :**:**: : . * ...* :**

**SO1985 PVQHNASNEIDVVDNDPWQQMLSNIMALPESTMSEPYIESSVTVSHRNIRYQIPRVFRQH 119**

**RSP1093 PVALSEG-------------SLASVMAQLDRQIQRP--APARRADPRAPAPLADYVGRRL 102**

**** . . *:.:** : :..* .: .. * * *:**

**SO1985 IALPWQQIGKVSRMRFDIDEKNTRASLLYIEALGEIPQHTHKGFELTLLLSGEFSDSTGN 179**

**RSP1093 EDVRWRTLGGGVRQAILPTGGEAIARLLWIPGGQAVPDHGHRGLELTLVLQGAFRDETDR 162**

**: *: :* * : :: * **:* . :*:* *:*:****:*.* * *.*..**

**SO1985 YVVGDFIILDGNTQHSPTTQTG--CLCYTVLDAPLYFTKGISKLLNPIGELIY 230**

**RSP1093 FGAGDIEIADQELEHTPVAERGLDCICLAATDAPLRFNSFLPKLVQPFFRI-- 213**

**: .**: * * : :*:*.:: * *:* :. **** *.. :.**::*: .:**

**Supplemental Figure S5** Clustal W alignment between the anti sigma factor ChrR (encoded by SO_1985) of *S. oneidensis* MR-1 and its orthologue in *Rhodobacter sphaeroides* to show the polypeptide sequence conservedness in the N- and C-terminal domains. The N-terminal domain could bind and sequester the ECF sigma factor RpoE2 (SO_1986) while the C-terminal domain could sense the singlet oxygen.

**ANA3 ---------GTGATCCAACTTCCCGCGCGCCACGTATT-GCT--CTGTAT----GCTTAT 44**

**MR7 ---------GTGATCCAACTTCCCGCGCGCCACGTATT-GCT--CTGTAT----GCTTAT 44**

**MR4 ---ACCTAAGTGATCCATTATTCAGCACTCTACGTATT-ACT--CCGCAT----GCTTAT 50**

**MR1 ---TTAACAGTGATCCATTATTCAAAGGGCCACGTATT-AGA--CAACAT----GCTTAT 50**

**W3181 --TTTTTCAGTGATCT-TTTTTTTGCCCATACCGTATTGACT--TAGCAA----GCAAA- 50**

**CN32 --TTTTTCAGTGATCT-TTTTTTTGCCCATACCGTATTGACT--TAGCAA----GCAAA- 50**

**OS195 -TCCTGTGAATGATCCTTTATTTAAGCCACTCCGTATTGATTGACAGTTT----GACAGT 55**

**OS185 -TCCTGTGAATGATCCTTTATTTAAGCCACTCCGTATTGATTGACAGTTT----GACAGT 55**

**OS155 -TCCTGTGAATGATCCTTTATTTAAGCCACTCCGTATTGATTGACAGTTT----GACAGT 55**

**OS223 -TCCTGTGAATGATCCTTTATTTAAGCCACTCCGTATTGATTGTAAGTTT----GACAGT 55**

**NCIMB400 TACATAACAGTGATCCGAACGCAAAAATGCCACGTATTAGTTAATGATACTGCAGCTTGT 60**

******* ****** ***

**-35 -10**

**ANA3 -CGAAT------------GTCGGCCCTGACGTAACATC-TTTTGTGATGTT----CAGA- 85**

**MR7 -CGACT------------GTCGGCCCTGACGTACCATC-TTTTGTGATGTT----CAGA- 85**

**MR4 -CGAAC------------GTCGGCCGTGAGGAAACATC-CTTTGCGATGTT----CTGA- 91**

**MR1 -CGAAC------------ATTGGAAGCGAAGCAGGATT-TGAAGCCATGTT----CGAA- 91**

**W3181 -CGAATTCAATGCGCTTTATTGGCGGAGAATCACGATC-----ATAGTGTT----CTAAA 100**

**CN32 -CGAATTCAATGCGCTTTATTGGCGGAGAATCACGATC-----ATAGTGTT----CTAAA 100**

**OS195 -AAAACTGGGC--------TCAGAAGTGAAACAAGCCT-CTCAATTTTGTCGTTACTGAT 105**

**OS185 -AAAACTGGGC--------TCAGAAGTGAAACAAGCCT-CTCAATTTTGTCGTTACTGAT 105**

**OS155 -AAAACTGGGC--------TCAGAAGTGAAACAAGCCT-CTCAATTTTGTCGTTACTGAT 105**

**OS223 -AAAACTGGGC--------TCAGAAGTGAAACAAGCCT-CTCAATTTTGTCGTTACTGAT 105**

**NCIMB400 TTGAGC-------------TCAGGATGGCCACAGCATAATGAGGTAATATT----TTATG 103**

*** * * * * * ***

**ANA3 --CT--GGATTG-----AACC-TTAAGTAAAGAGC-T-AATA 115**

**MR7 --CT--GGATTG-----AACC-TTAAGTAAAGAGC-TCAATA 116**

**MR4 --CT--GGATTG-----AACC-TTAAG-AAAGAGC-TCAATA 121**

**MR1 --CG--GGGTTG-----AACC-TTAAATGAAGAGC-TCAATA 122**

**W3181 CACA--GGATTT-----AATGATTAAGTTAAGAGC-AAAATA 134**

**CN32 CACA--GGATTT-----AATGATTAAGTTAAGAGC-AAAATA 134**

**OS195 CTCA--AACTTAGCACTAACGACTGGGATAAGAGCGAAAATA 145**

**OS185 CTCA--AACTTAGCACTAACGACTGGGATAAGAGCGAAAATA 145**

**OS155 CTCA--AACTTAGCACTAACGACTGGGATAAGAGCGAAAATA 145**

**OS223 CTCA--AACTTAGCACTAACGACTGGGATAAGAGCGAAAATA 145**

**NCIMB400 CTCAATGAGTT------ATTTATTGGAT-GCGGGT-GAAATA 137**

*** ** * * * * ******

**Supplemental Figure S6** Multiple alignment analysis on the nucleotide sequences upstream of the *rpoE2-chrR* cassette of *Shewanella* strains using Clustal W2 to identify the promoter motifs. The shadowed motifs are supposedly RpoE2-recognized elements for autoregulation.


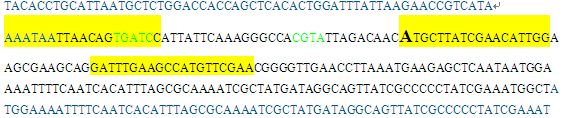


**-35**

**-10**

**PX-1986-1**

**PX-1986-2**

**PX-1986-3**


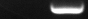

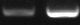

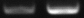

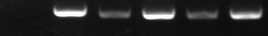


cDNA

gDNA

cDNA

cDNA

gDNA

gDNA

PX-1986-1

PX-1986-2

PX-1986-3


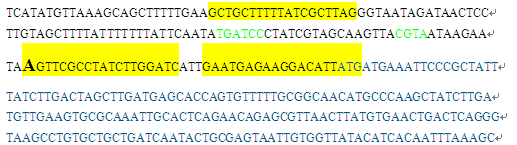


**-35**

**-10**

**PX-3349-1**

**PX-3349-2**

**PX-3349-3**


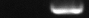

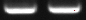

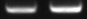

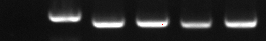


cDNA

gDNA

cDNA

cDNA

gDNA

gDNA

PX-3349-1

PX-3349-2

PX-3349-3

**Supplemental Figure S7** Mapping the transcriptional start site of *rpoE2* (SO_1986) and SO_3349 by using RT-PCR. Three forward primers (PX-1986-1,2,3, and PX-3349-1,2,3 highlighted in yellow color) and one reverse primer were used to map the start site of transcription. The cDNA and gDNA represent complementary DNA and genomic DNA as the PCR template, respectively. The PCR products were electrophoresis in the 1% agarose gels containing ethidium bromide and visualized by ultraviolet light and BioRad Image software.The star-indicated nucleotide is predicted to be the start site of transcription and the predicted -35 and -10 motifs are indicated in green color. The forward primers that target the sequence down-stream of start site could be used for PCR while the forward primers (PX-1986-1 and PX-3349-1) upstream of the start site failed to amplify the sequence from cDNA. These results further support the bioinformatics analyses of the RpoE2 recognized promoter motifs (Figure 9 in the main text) and are consistent with the RT-PCR results (Figure 10 in the main text).

| Predicted promoter sequence | Operon/transcription unit |
| --- | --- |
| -35 -10 |
| **TGATCC**TTGTACAAGAATGGTC**CGTA**ATAGTAT | SO_3386-SO_3374 |
| **TGATCC**CTATCGTAGCAAGTTA**CGTA**ATAAGAA | SO_3349-SO_3348 |
| **TGATCC**ATTATTCAAAGGGCCA**CGTA**TTAGACA | SO_1986-SO_1985 |
| **TGATC**AAATTCTGATGATGGTA**CGTA**ATGGTAG | SO_1987 |
| **TGATCC**TCACAGTGCTGCTATC**CGTA**ACGTTCA | SO_4169-SO_4170 |


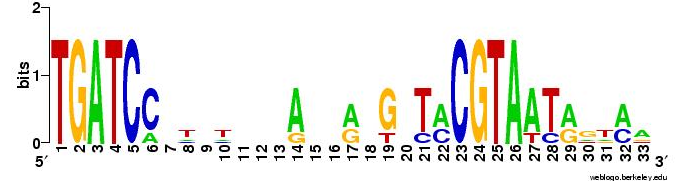


**Supplemental Figure S8** Sequence logos of the predicted RpoE_2_-dependent promoter motifs and some RpoE_2_-dependent operons in *S. oneidensis* MR-1.


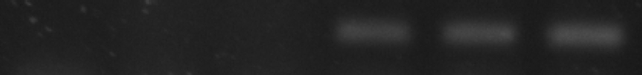


**C1**

***chrR***

**C2**

**C3**

**I1**

**I2**

**I3**


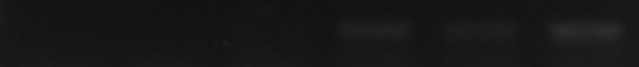


***rpoE2***


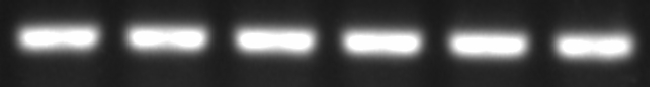


***16S rRNA***


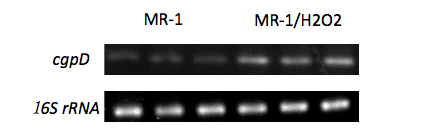


**Supplemental Figure S9** Induction of hydrogen peroxide on the transcription of *chrR,* *rpoE2* and *cgpD* genes. The *Shewanella* cultures were incubated to early exponential phase (OD_600_ was about 0.4) and the control samples for RNA extraction were taken. The hydrogen peroxide solution was added to bacterial culture to make the final concentration of 3 mM and the samples were also taken after two hours of induction. The left three lanes are the control samples and the right lanes are induced samples.


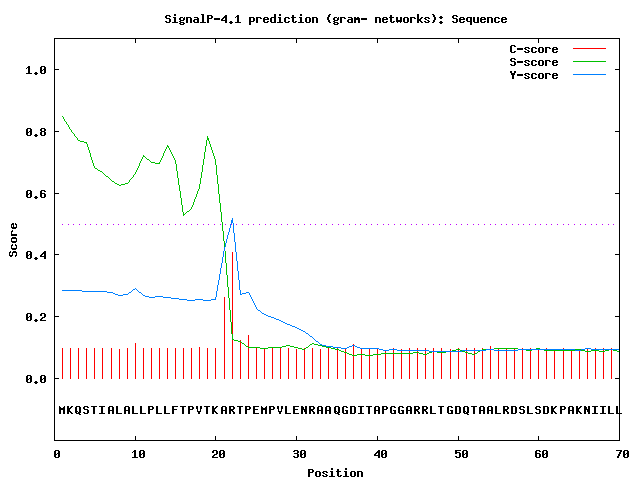


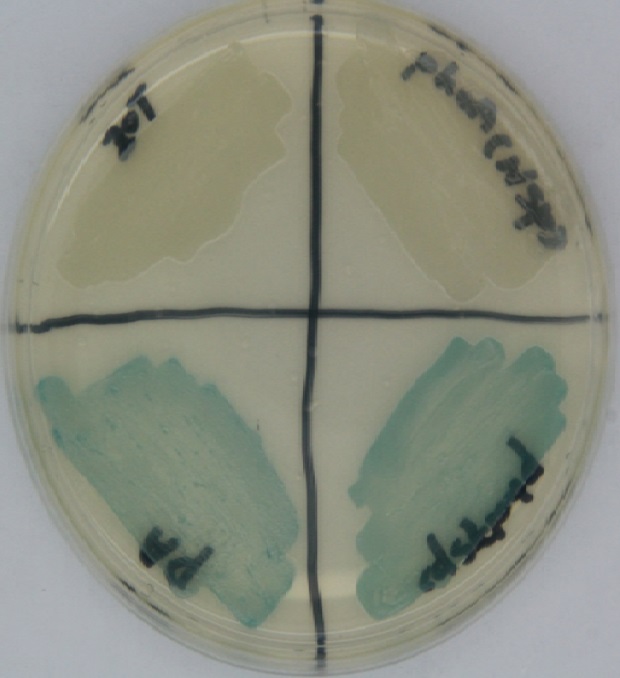


**pUCP20 vector**

**pUCP20-phoA(NSP)**

**pUCP20-pgpD-phoA fusion**

**pUCP20-phoA(wt)**

**Supplemental Figure S10** The alkaline phosphatase A (PhoA)-fusion assay demonstrates that the glutathione peroxidase protein PgpD is secreted into the periplasm as computationally predicted (upper panel) because the fused signal leader peptide of PgpD did resulted in the positive activity of PhoA (lower panel) in the *phoA* null strain DH5α. The upper panel is computational prediction of signal peptide for PgpD secretion by using the SignalP-4.1 software. The cleavage site of signal peptide is predicted to be located between amino acid residues 21 (A) and 22 (R). The nucleotide sequence encoding the PgpD signal peptide was fused with the *E. coli* *phoA* gene with deletion of sequence coding for original signal peptide by using cross-over PCR. The construct pUCP20-*phoA* expressing full-length PhoA (pUCP20-phoA(wt), wt stands for wild type) was used as positive control and the pUCP20-phoA(NSP) (NSP stands for no signal peptide) expressing the truncated PhoA without N-terminal signal leader sequence as negative control.

A

B

**Supplemental Figure S11** Genetic complementation analyses verifying the involvement of pgpD gene in oxidative stresses caused by hydrogen peroxide (A) and paraquat (B). The three tested strain are the empty vector-carried *Shewanella oneidensis* wild-type strain MR-1(MR-1) and the *pgpD* deletion mutant (MR-1ΔpgpD) as well as the mutant carrying the pHERD30T-pgpD construct (MR-1ΔpgpD +pgpD). All strains were grown in the LB broth containing 0, 0.5, 1, 2, 3, and 4 mM of paraquat or 0, 0.1, 0.3, 0.5, 0.7 and 1 mM of hydrogen peroxide and incubated at 28°C for 18 hrs.

**Supplemental Figure S12** Overexpression of *rpoE2* affected the bacterial growth under salt stress. The wild-type MR-1 strain carrying pHERD30T empty vector was used as control and 0.01% of L-arabinose(w/v) was added to the bacterial cultures of MR-1(carrying pHERD30T vector), MR-1ΔrpoE2 (carrying pHERD30T vector) and MR-1ΔrpoE2 overexpressing *rpoE2* (carrying pHERD30T-*rpoE2*) in the LB medium supplemented with 3% of sodium chloride (w/v).
